# Inhibition of N-Terminal Lysines Acetylation and Transcription Factor Assembly by Epirubicin Induced Deranged Cell Homeostasis

**DOI:** 10.1371/journal.pone.0051850

**Published:** 2012-12-14

**Authors:** Shahper N. Khan, Mohd Danishuddin, Bhavna Varshney, Sunil K. Lal, Asad U. Khan

**Affiliations:** 1 Interdisciplinary Biotechnology Unit, Aligarh Muslim University, Aligarh, India; 2 International Centre for Genetic Engineering and Biotechnology, New Delhi, India; Kyushu University, Japan

## Abstract

Epirubicin (EPI), an anthracycline antitumour antibiotic, is a known intercalating and DNA damaging agent. Here, we study the molecular interaction of EPI with histones and other cellular targets. EPI binding with histone core protein was predicted with spectroscopic and computational techniques. The molecular distance *r*, between donor (histone H3) and acceptor (EPI) was estimated using Förster’s theory of non-radiation energy transfer and the detailed binding phenomenon is expounded. Interestingly, the concentration dependent reduction in the acetylated states of histone H3 K9/K14 was observed suggesting more repressed chromatin state on EPI treatment. Its binding site near N-terminal lysines is further characterized by thermodynamic determinants and molecular docking studies. Specific DNA binding and inhibition of transcription factor (Tf)-DNA complex formation implicates EPI induced transcriptional inhibition. EPI also showed significant cell cycle arrest in drug treated cells. Chromatin fragmentation and loss of membrane integrity in EPI treated cells is suggestive of their commitment to cell death. This study provides an analysis of nucleosome dynamics during EPI treatment and provides a novel insight into its action.

## Introduction

The pharmacological treatment of cancer is often still unsuccessful. This is mainly due to the lack of discovery of new effective anticancer drugs, the severe side effects associated with conventional chemotherapy and the possibility of multidrug resistance, often induced by cytotoxic agent administration. Innovative ideas and new strategies are therefore needed in order to overcome these obstacles and to obtain the selective destruction of neoplastic cells. To this aim, optimal insights into the mechanisms of action at subcellular and molecular levels of antitumoural action and identification of their cellular targets constitute indispensable stages.

Epirubicin (EPI), an anthracycline antibiotic is the most common antitumor drug approved by the FDA for use in clinical oncology, especially in the treatment of acute carcinoma. Despite known cellular interactome of anthracyclines, the exact mechanism leading to tumor cell death is yet to be elucidated. Hence, the exploration and understanding of the process of their action has forced a reconsideration of the mechanisms whereby cells respond to anthracyclines. There is compelling evidence that cellular DNA is the primary target for this drug, but a simple intercalation model for the interaction of the drug with DNA is insufficient in explaining their mechanism of action in the cell. Some recent studies have shown that covalently binding agents disrupt the binding of transcription factors to their specific consensus sequences [1]. The ability of these adducts to prevent Tf-DNA binding in tumor cells infer that the sequence selectivity of a drug will determine which transcription factors are affected, and hence which genes are inhibited. This suggests that gene-specific inhibition may occur, depending on the sequence specificity of the drug adducts. In the cell nucleus, DNA is associated with a variety of proteins making a nucleoprotein complex called chromatin [2]. Interestingly, transcription in eukaryotic cells is influenced by the chromatin structure within which DNA is tightly packed with histones [3]. Histones undergo an array of modifications [4], [5], among which, acetylation is the most studied. [6], [7]. Acetylation of histones correlates with the transcription/transcriptional regulation of many genes. This opens new vistas for studying the affect of these drugs on histone acetylation. One possible physiological action of these drugs in tumor cells may involve the altered pattern of histone modification, more specifically histone acetylation which is supposed to play a crucial role in cellular proliferation and differentiation.

Anthracyclines exhibits a range of intracellular effects, the most prominent of which appears to be the induction of DNA damage and apoptosis. Depending on the organism, apoptosis involves a typical set of morphological events including chromatin condensation [8], [9], DNA and nuclear fragmentation, cell shrinkage, increased numbers of cytosolic vacuoles and formation of apoptotic bodies followed by phagocyte digestion [10]. In this study, we have tried to clarify basic cellular mechanisms mediating the biological action of EPI treatment and understanding the possible inflection of the drug action via molecular interactions with histone acetylation, DNA transcription and cell progression.

## Results

### Histone Core-drug Interaction

**Figure 1:**
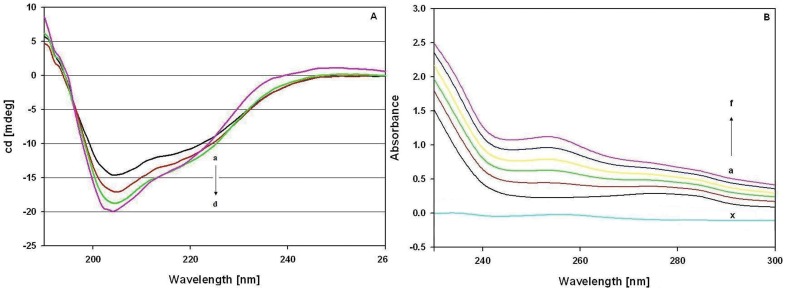
EPI interaction with core histone (A): The Far UV−CD spectra of core histones native (a); native+6.0×10^−6^M EPI (b); native+12.0×10^−6^M EPI (c) and native+24.0×10^−6^M EPI, (d). (**B**): Absorbance spectra of EPI, core Histone and core Histone–EPI system. Protein concentration was 12**×**10**^−^** ^6^ M (a). EPI concentration for drug–protein system was at 12**×**10**^−^** ^6^ M (b), 24**×**10**^−^** ^6^ M (c), 36**×**10**^−^** ^6^M (d), 48**×**10**^−^** ^6^ M (e) and 60**×**10**^−^** ^6^ M (f). A concentration of 12 ×10**^−^** ^6^ Μ EPI (x) was used for drug alone. The results presented are representative of at three independent experiments.

We employed circular dichroism (CD) to provide an insight into the structural alteration in the histone core upon drug treatment. In this work, the CD spectra of core histones exhibited one prominent negative band in the UV region around 208 nm and a small peak near 220 nm (Figure 1A, curve a), characteristic of an induced α-helical structure of histones [11]. The negative peak between 208 and 209 nm is probably an outcome of n→π* transfer from the peptide bond of α-helices [12]. Addition of the drug resulted in an increase in the negative peak (curve b) which increased further on addition of higher drug concentrations (curve c–d). Analysis of the native spectra (curve a) predicts α-helix  = 32.4%, β-sheet  = 23.2%, turn  = 15.1%, random coil  = 30.2%, whereas analysis of drug treated spectra (curve d) predicts α-helix  = 49.4%, β-sheet  = 22.1%, turn  = 13.6%, random coil  = 14.9%. The difference in percent structural component between the two curves suggests drastic structural perturbation upon EPI treatment. In another set of experiments, UV-Vis absorption studies were performed to further characterize the interaction complex of core histones and EPI. The intensity of the λmax was found to be increased with the increase in drug concentration (Figure 1B). Furthermore, a distinct blue-shift in λmax was also observed on treatment of protein with EPI. These two changes were indicative of a drug-protein complex formation [12], [13].

### Energy Transfer between Protein-drug Complex

**Figure 2:**
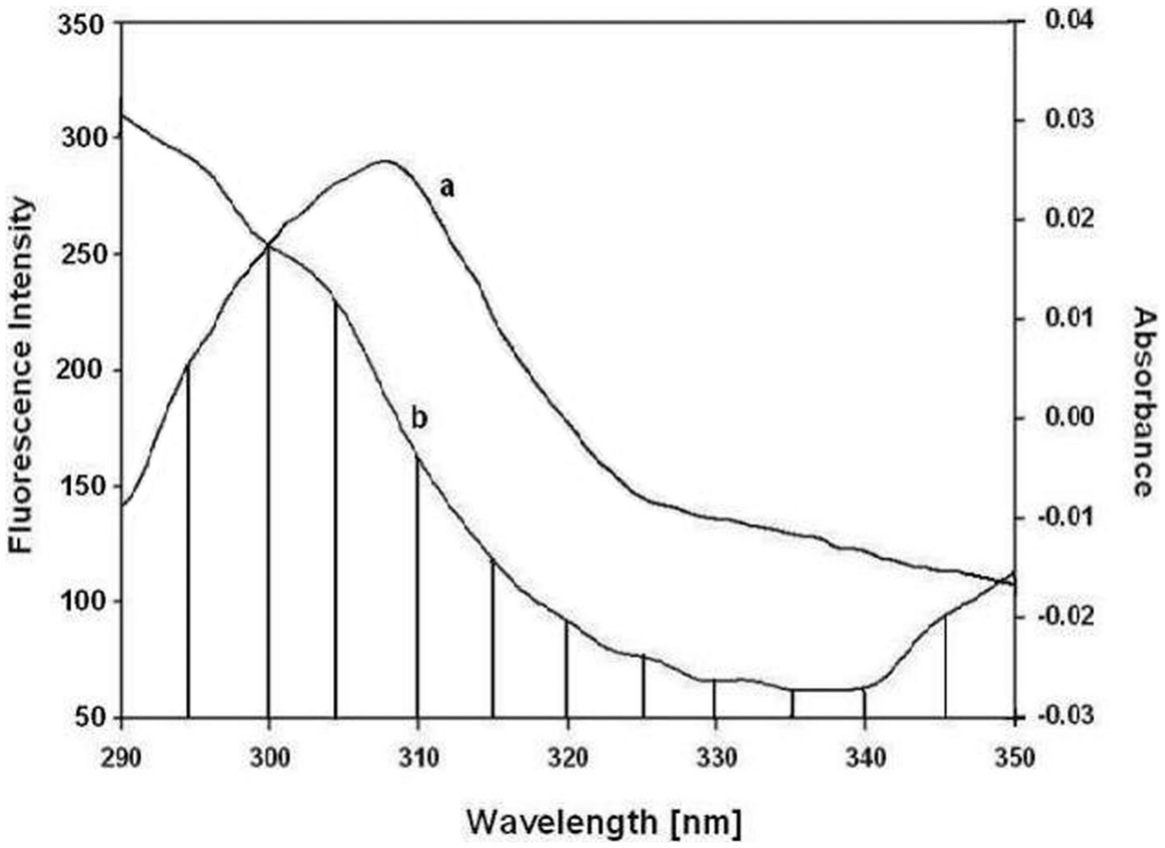
The overlap of the fluorescence spectrum of histone H3 (a) and the absorbance spectrum of the drug (b); [*c*(protein)/*c*(drug)  = 1∶1], for EPI.

The non-radiation energy transfer between the protein residue (donor) and the bound drug (acceptor) was determined using fluorescence resonance energy transfer (FRET). The overlap of the UV absorption spectrum of EPI with the fluorescence emission spectra of histone H3 is shown in figure 2. The distance between the donor and acceptor can be calculated according to Forster’s theory of dipole-dipole energy transfer [14]. In the present case, the values of *k* ^2^ = 2/3, *η*  = 1.336 and *Φ*  = 0.118 were used [15]. From Eq. (7) to (9), we were able to calculate the molecular distances and the efficiency of energy transfer to be *r*  = 1.86, *R*_0_ = 1.44 and *E* = 0.10, respectively. As the maximal critical distance, the *R*_0_ range is from 5 to 10 nm [16] and the maximum distance between donor and acceptor for *r* is in the range of 7–10 nm [16] for non-radiation energy transfer to occur. Hence, the values of *R*_0_ and *r* for EPI suggested that non-radiation transfer occurred between the drug and the protein.

### Drug Induced Inhibition of Histone Acetylation

**Figure 3:**
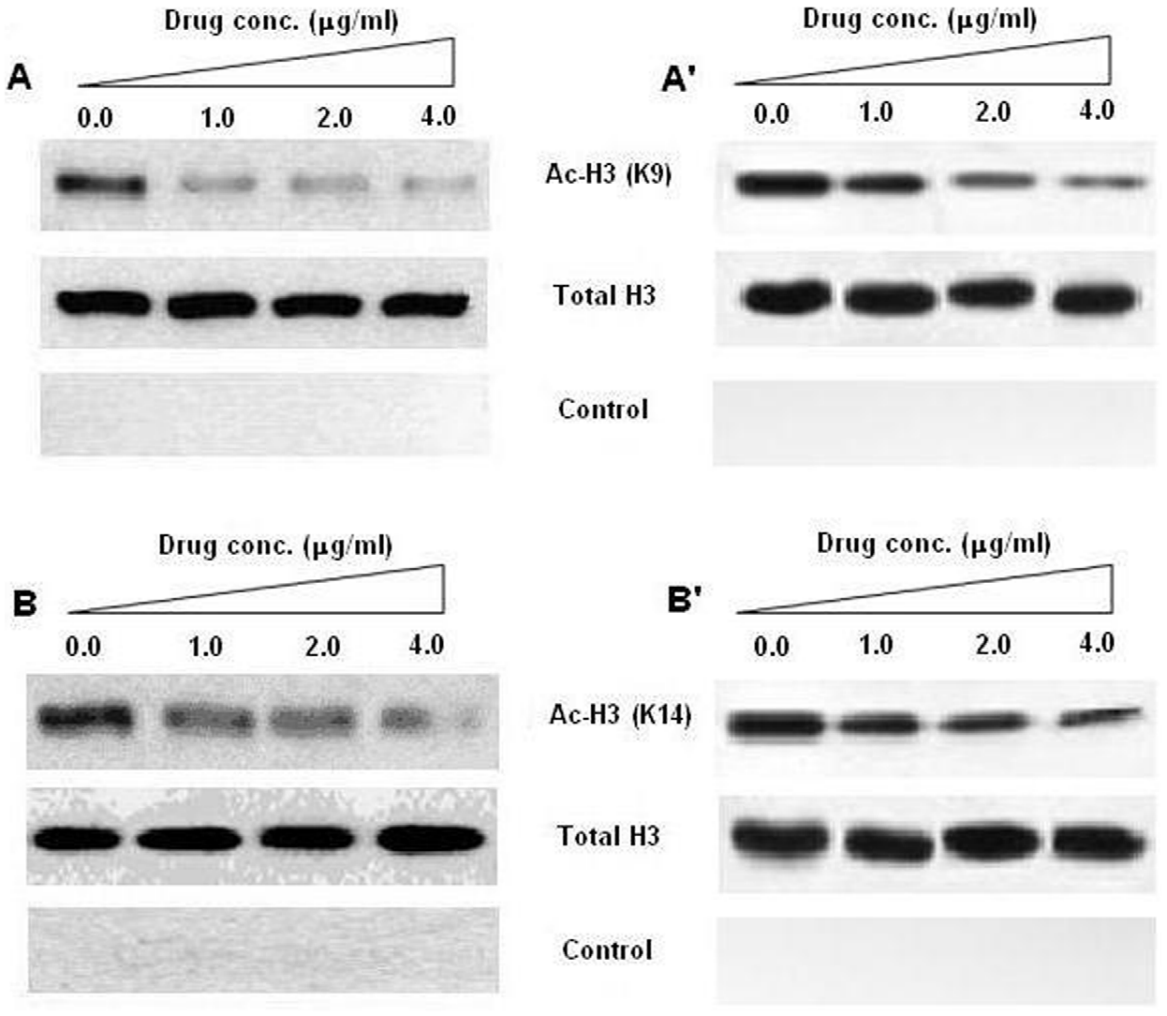
Representative, western blot analysis of histone H3 (Lysine 9/K9 and Lysine 14/K14) acetylation. Yeast and HEK293 cells were treated with different concentration of EPI, to analyze the concentration dependent response of the drug on H3 acetylation pattern. Figure represents the effect of EPI treatment on acetylation pattern in yeast (panel A and B) and HEK293 (A’ and B’) cells, respectively. Samples with no antibody added were used as a negative control (control) and total H3 was used as the input control for K9 and K14, respectively.

Along with its direct binding with the protein, EPI can affect the posttranslational modification of histone. Hence, we evaluated the affect of EPI-Histone core complex on histone acetylation *in vivo*. We analyzed the acetylation of histone H3 K9/K14 specifically to evaluate the drug induced effect in HEK293 and yeast cells (AH109). Results showed a serial drop in acetylation pattern with increasing concentration of the drug (Figure 3). The results were further confirmed in a cancerous line SKM-1 (Figure S3).

### Histone (H3) Binding with EPI

**Figure 4:**
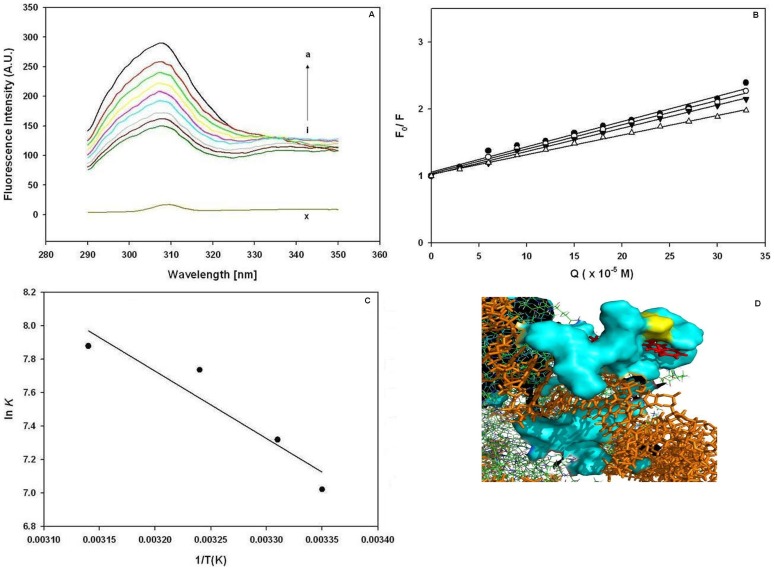
Binding analysis of histone H3 with EPI (A): Effect of EPI on fluorescence spectrum of Histone H3 (*T*  = 298 K, pH 7.40, λex  = 280 nm). a–i, [Protein]  = 3.0 · 10^−5^ M, [Drug]  = 0.0, 3.0, 6.0, 9.0, 12.0, 15.0, 18.0, 21.0 and 24.0×10^−5^M, respectively. Curve x, shows the emission spectrum of EPI alone. (B): Stern–Volmer plot, for the binding of histone H3 with EPI. The plot represents the binding of the drug at 298 (○), 302 (•), 306 (▾) and 310 (Δ) K. (C): The Vant hoff plot for the binding of histone H3 with EPI to evaluate the thermodynamic parameters. The results presented are representative of at three independent titration experiments with ±SD. (D): molecular modeling of histone H3-EPI complex. Drug and DNA represented as stick model in red and orange color, respectively. Histone H3 shown in surface filled model cyan and lysine 9 depicted as yellow in the vicinity of drug binding pocket.

The above results were suggestive of the interaction of EPI with the histone H3. Hence, we further evaluate the binding of this drug specifically with histone H3. The intrinsic fluorescence of histone H3 was recorded with increasing concentration of EPI in the range of 290–350 nm. Figure 4A, illustrates proportionate reduction in the intrinsic fluorescence of the protein, more effective quenching of the chromophore molecule fluorescence was observed at higher concentration of EPI. The shift in the emission maximum of the protein from 307 to 309 nm was also observed on EPI binding (Figure 4, A). The binding isotherm (Figure 4, B) shows the drug concentration dependent quenching of intrinsic fluorescence of protein. The procedure of quenching was further resolved from the values of bimolecular quenching rate constants *K*q, found to be of the order of 10^14^L mol**^−^** ^1^ s**^−^** ^1^, which was greater then the maximum limiting diffusion constant K_dif_ of the biomolecule (*K*_dif_  = 2.0×10^10^ Lmol**^−^** ^1^ s**^−^** ^1^) [15], suggesting the static type quenching procedure. The *K*_SV_ values decreased with increase in temperature (Table 1), which indicates that the probable quenching mechanism of histone H3 fluorescence by EPI is a static type. The plot of log (*F*_0_ −*F*)/*F* versus log [Q] was used to determine the values of *K* and *n* at 298, 302, 306 and 310 K (Table 1), which suggested that the binding strengthens with increase in temperature. Meanwhile, from the data of *n* it may be inferred that there is one (≈1) site of binding for this drug.

**Table 1:** Effect of temperature on quenching constants of histone H3–EPI Complex.

T (*K*)	*K*_SV_ ×10^−3^(Lmol^−1^)	*K*q ×10^−14^(Lmol^−1^s^−1^)	*R* ^2^	*K*×10^−4^(mol^−1^)	*n*
298	3.78±0.06	3.78±0.06	0.983	0.11±0.22	0.84
302	3.63±0.04	3.63±0.04	0.995	0.15±0.06	0.89
306	3.48±0.12	3.48±0.12	0.996	0.22±0.14	0.94
310	2.92±0.15	2.92±0.15	0.996	0.26±0.26	0.95

### Thermodynamic Analysis of Drug-protein Binding

The thermodynamic parameters were analyzed by Van’t Hoff plot (Figure 4, C). Results (Table 2) suggest that the process is entropically driven. The positive entropy changes occur because the water molecules that are arranged in an orderly fashion around the ligand and protein acquire a more random configuration as a result of hydrophobic interactions. The negative sign for Δ*G*° indicates the spontaneity of the binding of the drug with histone H3. For EPI complex with histone, the main source of binding free energy is derived from a large contribution of term Δ*H*° as compared to the Δ*S*° factor, so that the electrostatic interaction dominate, but the hydrophobic interactions can not be ignored in formation of the drug-protein complexes. Further, molecular modeling studies were employed to predict the precise binding pocked for EPI on histone H3. Best energy results has placed drug near N-terminal region of histone H3 sharing the binding site with bound DNA (Figure 4D). The residues involved in the binding of EPI in the binding cavity are predicted and their respective molecular distances from the bound drug have been evaluated with the Getnears (Table 3). The specificity of the binding was also evaluated by docking the structurally similar drugs DOX and DNR from anthracycline group at EPI binding site (Figure S4). The GOLD fitness score for all three binding interactions was 54.65, 53.87 and 52.32 for EPI, DOX and DNR respectively.

**Table 2:** Thermodynamic parameters for histone H3-EPI binding.

T (*K*)	Δ*G*° (KJmol^−1^)	Δ*H*° (KJmol^−1^)	Δ*S*° (KJmol^−1^ *K* ^−1^)
298	–17.48±0.35	33.47±0.14	0.171±0.21
302	–18.17±0.35	33.47±0.14	0.171±0.21
306	–18.85±0.35	33.47±0.14	0.171±0.21
310	–19.54±0.35	33.47±0.14	0.171±0.21

**Table 3:** Molecular docking analysis of the EPI binding on histone octamer complex, predicting atoms involved and estimated distances between the protein and ligand atoms.

Ligand atom	Receptor atom	Distance(Å)
EPI C29	DA 231	3.79
EPI C22	DA 231	3.5
EPI C29	DC 230	3.6
EPI C29	DC 230	3.54
EPI C30	DT 64	3.59
EPI C30	DT 64	3.72
EPI O8	DT 64	2.42^H^
EPI C19	ARG 40	3.25
EPI C20	ARG 40	3.89
EPI C19	ARG 40	3.56
EPI C21	GLY 33	3.8
EPI C31	ALA 31	3.29
EPI C28	ALA 31	3.84
EPI C27	ALA 31	3.05
EPI C24	ALA 31	2.89
EPI C17	ALA 31	3.59
EPI C31	ALA 31	3.57
EPI C27	ALA 31	3.83
EPI C39	ALA 29	3.83
EPI O9	ALA 29	2.50^H^
EPI N12	LYS 37	2.77^H^
EPI O2	ARG 40	2.43^H^

H -hydrogen bonding.

### DNA Binding Specificity of EPI

**Figure 5:**
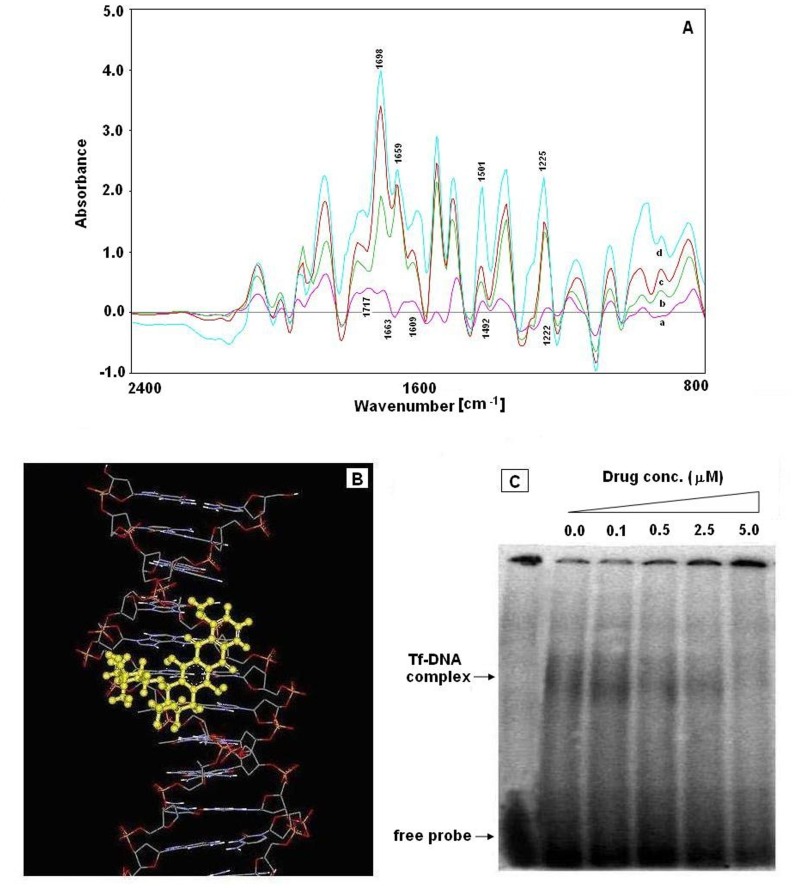
EPI binding hinders DNA-protein interaction (A): FTIR difference spectra of DNA in the absence and presence of increasing concentration of EPI. Curve a-d corresponds DNA (3.0 mM)+drug (a) 0 µM; (b) 3 µM; (c) 30 µM and (d) 300 µM. (**B**): Molecular modeling of DNA binding with EPI. (C): Gel retardation assay of inhibition of binding by octamer proteins with increasing concentration of EPI. The octamer binding probe (H2B gene) was exposed to 0.1–5.0 µM of EPI for an incubation period of 12 h. Control samples were incubated in the absence of drug in the reaction buffer. The reacted probe was exposed to nuclear extract from HEK293 cells. Electrophoretically retarded bands denote protein-DNA complexes due to Oct proteins present in the nuclear extract.

The spectral changes (intensity and shifting) of several prominent DNA signals in-plane vibrations at 1717 cm**^−^** ^1^ (G, T), 1663 cm**^−^** ^1^ (T, G, A and C), 1609 cm**^−^** ^1^ (A, C), 1492 cm**^−^** ^1^ (C, G) and 1222 cm**^−^** ^1^ (PO_2_ asymmetric stretch) [17], [18], were monitored at different drug concentrations by employing Fourier transform infrared (FTIR) spectroscopy**.** The increased intensity of DNA in-plane vibrations at 1717 (G), 1663 (T), 1609 (A), and 1222 cm**^−^** ^1^ (PO_2_ asymmetric stretch) upon EPI interaction (Figure 5, A) were suggestive of intercalative binding, which also corroborates the hyperchromism and downward shift of absorption maxima depicted by electronic absorption spectra of DNA-drug complex (Figure S1). The major shifting of the band at 1717 (G) to 1698 and 1726 cm**^−^** ^1^ on EPI treatment is indicative of drug intercalation mainly into the G–C base pairs (Figure 5, A). However, the thymine band at 1663 cm**^−^** ^1^ exhibited no shifting upon drug complexation. Since adenine band at 1609 cm**^−^** ^1^ showed an increase in absorbance with almost no shift in the peak, it was difficult to determine the nature of the drug interaction with A–T bases (Figure 5, A). At higher drug concentrations, a major increase in intensity of the guanine band at 1717 cm**^−^** ^1^ was accompanied by the shifting of this vibration, which is indicative of EPI binding with guanine bases. This illustrates the higher affinity of this drug for G-C base pairs. The shifting of the PO_2_ asymmetric band at 1222–1224 cm**^−^** ^1^ with increase in intensity of this vibration illustrates the interaction of this drug with the backbone PO_2_ groups, implicating its binding near phosphate backbone. Molecular docking studies were employed to establish the molecular interactions between DNA and EPI. Although the crystal structure of the complex can represent specific details of the interactions, general observations may be obtained from docking studies. The docked structure as shown in Figure 5(B) suggests that EPI could bind to DNA by interacting with the bases and the phosphate backbone. The binding site of the drug is 3 base pairs long. Figure 5 (B) also illustrates the interaction of the aromatic ring and the side chains of the drug moiety with a base pair and phosphate back bone of DNA, which helps in better ‘anchoring’ and stabilization of the drug-DNA complex. The analysis of the docking results further revealed the binding of the drug at the G–C rich region. As the DNA template used was comprised of AT and GC in 1∶1 ratio, this suggests some GC specificity toward drug binding. This corroborates with our previous results of higher G–C affinity of EPI from FTIR.

### EPI Inhibits Transcription Complex Formation

Ability of this drug to inhibit transcription factor (Tf)-DNA complex formation was analyzed/tested by EMSA. The octamer motif is important for promoter activation mediated through generally expressed Oct-1 protein. The octamer element is also a target-binding site for octamer factors [19]. Hence, we chose octamer consensus motif for EMSA. Initial studies were performed to establish whether EPI binding would prevent octamer protein binding to their consensus motif. The crystal structure of Oct-1 bound to *ATGCAAT* revealed that the specific domain of Oct protein makes contacts mainly with GCs within the sequence [20]. The predicted specificity of the drug from above studies, which is also supported by the tetracyclic structure, suggests that binding of this drug at GC sites would pose a direct blockage to Tf-DNA interaction. Figure 5 C, shows that EPI inhibits octamer binding with the consensus motif in a concentration-dependent manner. To avoid any artefacts, the binding reaction was carried out in the presence of the non-specific oligo, poly (dI-dC). Overall, the inhibition of binding of octamer proteins correlates well with the formation of characteristics anthracycline induced adducts. Although these trends are quite clear and highly reproducible, there was some variation in the absolute level of formation of octamer protein-DNA complexes which could be explained due to the varying concentration of transcription factors present in individual nuclear extracts [21].

### Drug Induced Gene Repression

The DNA-binding sequence for Gal4p (UASgal) [22] contains two palindromic CGG repeats, which are also binding sites for EPI. We thus explored the effect of the drug on the ability of Gal4p to activate transcription.

**Figure 6:**
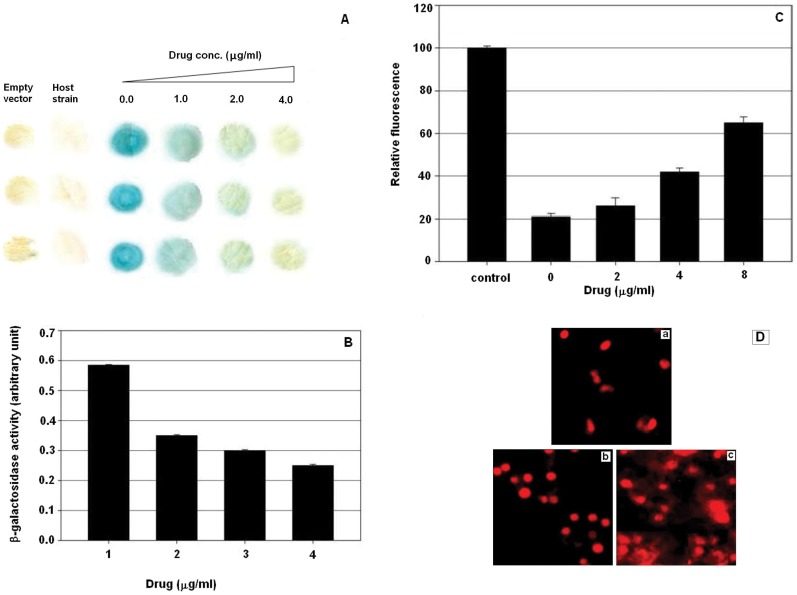
Effect of EPI on cellular dynamics (A): Representative spots for colony lift assay. Expression of *lac Z* gene in yeast two hybrid system in the absence and presence of increasing concentration of epirubicin (EPI), show as a function of β-galactosidase activity. Colony of host strain containg empty vector depicts the untransformed cell used as the blank for background/innate colour of the cells. (**B**): Effect of EPI on β-galactosidase activity of EPI treatment. Representative plot of the enzyme activity in absence and presence of drugs, correlating drug induced gene repression. The values plotted here are corrected by subtracting the blank from the studied samples and are the mean ± SD values of three independent experiments. (**C**): Representative plot of relative fluorescence intensities of cells on EPI treatment. The plot depicts the fluorescence intensities of isopropanol treated control and the tested samples in the absence and presence of increasing concentration of the drug. Culture conditions are explained in experimental section. Results from three independent experiments reported as means ± SD. (**D**): Confocal images of yeast cells in the absence and presence of EPI. Panel a, represents the untreated control cell grown in YPD media for 24hrs. Panel b & c, represents the cells grown in the presence of the 4 and 16**×**10**^−^** ^6^ M drug for 24 hrs, respectively.

Colony lift (filter) assay was used to determine the effect of EPI on the expression of a *lac Z* gene fused with GAL4 DNA-binding domain. The effect of drug on the gene expression was determined by analyzing the blue colour due to β-galactosidase activity. Figure 6(A), shows the representative colony lifts in the absence and presence of the tested drug. The results showed the drastic diminution of the colour on treatment of cells with 4 µg/ml of the EPI, which was indicative of drug induced gene repression.

Furthermore, we quantified the effect of EPI on β-galactosidase activity by performing liquid β-gal assay. The yeast cell suspension culture was treated with different concentration of the drug followed by harvesting and washing with the PBS to eliminate the possibility of the drug caused enzyme inhibition. The negative (untransformed strain) and positive (transformed strain without drug treatment) controls were set to accurately evaluate the enzyme activity. Results are represented as mean relative β-galactosidase activity (Figure 6, B). The data illustrates a marked reduction in the enzyme activity in dose dependent manner.

### EPI Induced Membrane Permeability

Since EPI affects cell growth and viability (data not shown), which might be related to the accumulation of dead cells on drug. Therefore cell membrane integrity was investigated in this experiment. HEK293 cells treated with or without EPI were stained with PI and scanned for fluorescence emission after excitation at 490 nm. Figure 6(C), illustrates the dose dependent effect of the fluorescence of PI. The figure depicts the increase of fluorescence with increasing drug concentration. This increase in fluorescence corresponds to the increase in cell injury that is caused by repairable membrane damage in the cellular permeability on treatment of cells with the drug. During extended cultivation the control cells retained impermeability to PI, which further demonstrated that the drug induced permeability in the treated cells was not due to the developmental stages of the cell.

### Drug Induced Chromatin Fragmentation

We estimated the DNA content of the cells grown in the presence and absence of EPI. Yeast cells stained with DNA binding dye were visualized by confocal microscopy (Figure 6, D). EPI treated cells when compared with the drug treated cells were found with compacted DNA content and further addition of EPI lead to DNA fragmentation. The focused fluorescence suggested chromatin condensation and dispersed fluorescence signals indicated some fragmentation in the EPI treated cells (Figure 6, D).

### EPI Affects Cell Cycle Distribution

**Figure 7:**
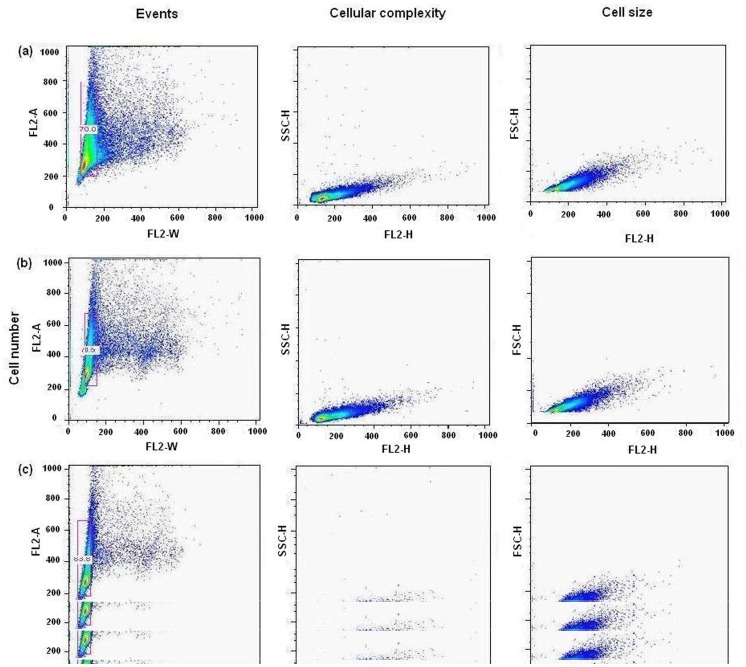
Representative cytograms of the yeast cells grown with and without the treatment of EPI. Panel a, depicts the untreated cells used as the control. Panel b & c, represents the cells grown with increasing concentration of the drug. Respective bivariate dot plots of cellular complexity (side scatter; SSC-H vs FL2-H) and cell size (forward scatter; FSC-H vs FL2-H) are reported on the right side of their respective cytogram.

To determine the effect of drug treatment on cell cycle redistribution, yeast cells were treated with IC_12.5_ and IC_25_ doses for 24 h and analyzed by flow cytometry. Figure 7, represents the events detected by flow cytometry in the absence and presence of increasing concentration of EPI. The cytograms reported on the right hand side of the profiles represent an analysis of their respective cell complexity (with side scatter) and cell size (with forward scatter). The complexity of the cells seems to decrease on increasing concentration of EPI as compared with the untreated cells. This suggests that cells are arrested at the initial phases of cell cycle, thereby minimizing the cellular complexity. Comparable cell size, found in the control and EPI treated cell groups rule out the presence of cell debris in the analyzed cell population. The frequency histograms (Figure 7) from FACS analysis illustrate a dose-dependent arrest in yeast cells. Table 4 represents the phase distribution of cells after treatment with EPI, which suggests that the highest number of cells were found in G1 and S phase. There was no evidence of apoptotic peaks (left of the Go-G1 peak) with any of the EPI concentrations. The FACS analysis was repeated in triplicate, and the data revealed a statistically significant cell-cycle arrest with the EPI (*P*≤0.005).

**Table 4:** Effect of EPI treatment* on cell cycle distribution of yeast cells.

Drug conc. (µg/ml)	(% cell distribution)		
	G1	S	G2
0	38	28	34
2.5	42	40	18
5	52	44	4

* Cells were treated for 24 hrs with EPI.

## Discussion

The interaction of antitumour drugs with chromatin has been the subject of many reports, which have consistently shown the binding of these drugs to chromatin [23], [24]. Chromatin consists of DNA, histones and a plethora of different protein complexes that assist with the dynamic changes occurring during DNA replication, cell-cycle progression, regulated-transcriptional and post-transcriptional events, DNA repair and recombination. Hence, the eukaryote cell chromatin is thought to be the main target of action of anthracycline drugs [25]. Despite these studies, how the drug interferes with these regulatory events is still unclear. In this work, we investigated the interaction of epirubicin (EPI) with novel targets in chromatin and its interference in regulation of basal transcription and cell proliferation.

The interaction of histone core with EPI (Figure 1 and 2) conclusively shows that drug binding with protein led to structural perturbation of the protein. Forster theory predicted the close proximity and the non-radiation energy transfer between EPI and Histones [26]. Furthermore, reduction in the histone H3 K9/K14 acetylation (Figure 3) shows the drug induced inhibition of acetylation. Though such reduction in acetylation was not found with other histone H3 lysines (Figure S5). Histone acetylation at the N-terminal lysines is associated with the transcriptional activation of specific genes [27]. Also the role of N-terminal tails in anticancer drugs with nucleosomal DNA has been discussed recently by Mir and Dasgupta [28]. Hence, the inhibition of acetylation of histone correlates with transcriptional activity in many genes, allowing DNA to remain in the compact state and thus not providing access for transcription factors to bind on their targeted promoters. Changes in the level of histones H3 acetylation at specific lysine residues in several malignancies [29], [30], shows the importance of specific patterns of histone acetylation at N-terminal lysine residues for the final outcome of gene regulation.

Quenching of intrinsic fluorescence of histone H3 (Figure 4) implies the changed solvent accessibility of the protein induced by drug binding in the vicinity of aromatic fluorophore. The quenching of the protein fluorescence clearly indicated that the binding of the drug to protein has changed the microenvironment of the fluorophore. The thermodynamic parameters are also in agreement with molecular modeling studies, depicting the role of electrostatic and hydrophobic contacts in stabilization of protein-drug complex. This binding is prominently targeted to the histone H3. Hydrogen bonding acts as an “anchor”, intensely shaping the 3D spatial position of EPI in the binding pocket and facilitates hydrophobic interactions of the dihydroxyanthraquinone rings with the side chain of (lysine, arginine, proline and histidine residues) protein. It is worth mentioning that the docking procedure placed the drug molecules within the vicinity of N-terminal lysines (histone H3 K9/K14), implicating its interference in histone modification, which corroborates with reduction in acetylation levels *in vivo*. The GOLD fitness scores predits the best binding near N-terminal lysine with EPI than DOX or DNR. But similar effects with structurally similar anthraclyycines cannot be fully denied.

The purpose of the DNA binding studies was to examine the preferential binding site of EPI and inhibition of Tf-DNA complex induced by this interaction. The major increase in the guanine and cytocine vibrations and their respective shifts illustrates the higher affinity of interaction between GC bases and EPI. Molecular modeling predicted EPI major groove binding as the lowest energy conformation. From the gel shift assays shown in figure 5D, it is clear that micromolar levels of EPI form a sufficient number of adducts with the wild-type octamer sequence to inhibit the binding of Oct protein. The presence of Oct-1 protein was confirmed by supershift assay (Figure S2). Overall, the results from this study indicate that the binding of EPI to DNA does have a potential inhibitory role *in vivo.* Though, it is questionable whether the extent of inhibition of octamer protein binding facilitated by EPI would be significant enough to lead to detectably altered regulation patterns of specific cellular proteins, but it is possible that the effects of this interaction could be further magnified when the complex interactions of proteins required in transcription initiation are considered.

The inherent complexity of transcription regulation and gene expression in eukaryotes makes it difficult to correlate the direct effect of EPI on *in vivo* systems from indirect effects of inhibition of histone acetylation or Tf-DNA complex formation. Therefore, we used Gal4 based yeast two hybrid system to examine the determinants of the particular cytotoxic effect. Our data (Figure 6, A and B), shows the concentration dependent inhibition of Gal4p function and resulting lac *Z* gene differential expression with low concentrations of EPI, which was found to be statistically significant when compared to untreated control (*p*<0.05). Relatively low concentrations (1–4 µM) of EPI prevented the growth of yeast cells in the medium. EPI induced concentration dependent membrane permeability (Figure 6, C), is suggestive of the drug effect on cellular integrity. Membranes have been demonstrated to play a crucial role in the cytotoxicity induced by drugs, as they are important structural and functional components of all cell types. They have been shown to be one of the important targets of action of the anthracyclines [31]. Regulated progression through the cell cycle and its checkpoints is essential for a normal cell proliferation [32]. Our results (Figure 7) show that EPI promotes cell-cycle arrest and growth inhibition by forcing G1 phase arrest, and that arrest of S phase suggests the blockage of synthetic phase, thereby minimizing the synthesis and doubling of metabolically important constituents required for proliferation and sustenance of cells. By promoting cell cycle arrest, DNA fragmentation, membrane permeability and subsequent growth inhibition, cells possibly undergo a check for apoptosis [33].

**Figure 8:**
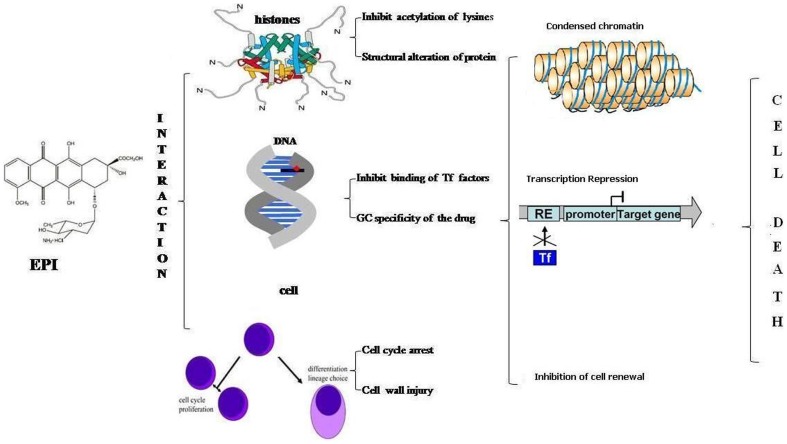
Model representing the novel targets of EPI action. EPI interaction with histone inhibits the acetylation of lysine, resulting in more compact and transcriptionally repressed chromatin. EPI binding with promoter elements inhibit the binding of transcription factors and may prevent the formation of pre-initiation complex. Further drug induced cell injury via cell permeability DNA fragmentation and cell cycle arrest halt cell renewal and mark cell for apoptosis or cell death.

This study established a mechanistic model of EPI action. The results implicate the hindrance of the acetylation process of lysine by binding near the N-terminal of histone core complex. EPI interaction with histone acetylation at respective levels might cause a blockage of its acetylation assisted regulation. The specificity of DNA drug binding provides selectivity towards its actions. Membrane permeability, DNA damage and cell cycle arrest signal the onset of apoptosis in drug treated cells. Thus this study for the first time correlates the antiproliferative activity of EPI with histone acetylation and illustrates the obstruction of the Tf-DNA complex (Figure 8). The establishment of a mechanistic model of EPI binding will aid in the development of small molecules with the ability to interfere with the expression of specific genes in living cells and may improve the current therapies for human diseases [34], [35], as they may block specific pathways necessary for cell growth.

## Materials and Methods

### Materials

Calf thymus histone (Cat # 10223565001) and Histone H3 (Cat #11034758001) was purchased from Roche Applied Science. Before the reconstitution experiment, histones proteins were analyzed by SDS gel. The histones were reconstituted in 2.0 M NaCl and diluted in 10 mM phosphate buffer of pH 7.4 just before every experiment [36]. Epirubicin (EPI) was procured from Sigma-Aldrich Chemicals. Anti-acetyl histones H3 K9/K14 were purchased from Upstate Biotechnology, anti-histone H3 antibodies were from Santa Cruz Biotechnology.

### Drug-histones Binding

To obtain the correct time of incubation, fixed concentration of histone protein was incubated with various concentrations of EPI. Free drug and histones prepared in the same buffer and incubated along with the drug-histone samples under the same conditions were used as controls where required.

### Circular Dichroism (CD)

Circular dichroism (CD) measurements of histone core in the presence and absence of EPI were performed in the far-UV (200–250nm) region on a Jasco-J820 spectropolarimeter. All the spectra were substracted with appropriate control spectra (buffer+drug) from the spectra of samples (protein+drug+buffer) and were smoothed within permissible limits by the inbuilt software of the instrument. The molar ratios of histones to drug concentration were 1∶0, 1∶2 and 1∶4. The CD results were expressed in terms of mean residual ellipticity (MRE) in deg · cm^2^ · dmol^–1^ according to the following equation:
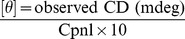
(1)


Where [θ] is mean residual ellipticity, *C*p is the molar concentration of the protein, *n* the number of amino-acid residues and *l* is the path length. The instrument was equipped with software based on the Yang equation [37] to calculate the secondary structure content in the protein.

### Absorption Spectroscopy

The UV measurements were recorded on a Shimadzu double beam spectrophotometer model-UV 1700 using a cuvette of 1 cm path length. Absorbance values of protein in the presence and absence of EPI were in the range of 250–300 nm and protein concentration was fixed to 12 µM while the drug concentration was varied from 12 to 60 µM. Baseline was corrected with suitable control solutions.

### Molecular Modeling Studies

Molecular docking simulations of histone octamer, and DNA interactions with the EPI were performed with the DOCKv5 program. The crystal structures of the proteins were obtained from the Brookhaven protein data bank (PDB: 1EQZ, 453D). The two dimensional (2D) structure of epirubicin (EPI), doxorubicin (DOX) and daunorubicin (DNR) was retrieved from the Pubchem (pubchem.ncbi.nlm.nih.gov). Genetic algorithm was implemented in DOCKv5 to calculate the possible conformations of the drug that binds to the proteins. The genetic algorithm parameters used were: Population size-100; Number of Islands-5; Niche size-2; Selection pressure-1.1; Migrate-2; Number of operators-100,000; Mutate-95; Crossover-95. During the docking process, a maximum of 20 different conformations were considered for the drug. The conformer with the lowest binding free energy was used for further analysis. The binding energy of docked complexes was calculated using X-Score [38]. The residues involved in hydrogen bonding and hydrophobic interactions were calculated using GetNeares, a tool available with DOCKv5.

### Fluorescence Spectroscopy

Fluorescence measurements were made on a Luminescence spectrometer, model LS55 (Perkin Elmer). The fluorescence quenching of histone intrinsic fluorescence at increasing molar ratios of the drug to protein was recorded in the wavelength range 290–350 nm after exciting the protein solution at 278 nm, using 3 nm/3nm as slit widths. The protein concentration was fixed at 3.0**×**10**^−^** ^5^ M and the drug concentration was varied from 3.0 to 34.0**×**10**^−^** ^5^ M. To evaluate the effect of temperature on drug-histone binding, fluorescence was recorded at four different temperatures i.e. 298, 301, 306 and 310 *K*. The optical densities of the samples were lower than 0.05 at the excitation wavelength to avoid inner filter effect.

### Analysis of Binding

To elaborate the fluorescence quenching mechanism the Stern-Volmer equation was used for data analysis:
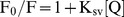
(2)where *F*_0_ and *F* are the steady-state fluorescence intensities in the absence and presence of quencher, respectively. *K*_SV_ the Stern–Volmer quenching constant and [Q] is the concentration of quencher (EPI). The bimolecular quenching rate constants, *K*q were evaluated using the equation:
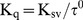
(3)where τ_0_ is the lifetime of protein without the quencher. The average value of fluorescence lifetime used was about 10−8 [39]. When ligand molecules bind independently to a set of equivalent sites on a macromolecule, the equilibrium between free and bound molecules is given by the equation [40]:
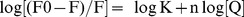
(4)where K and n are the binding constant and the number of binding sites, respectively. The values of the binding constant and binding site were fitted into following equation to evaluate the thermodynamic parameters:



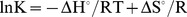
(5)

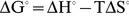
(6)where *K* and *R* are the binding constant and gas constant, respectively.

### Energy Transfer between the Drugs and the Protein

The absorption spectrum of EPI (3.0**×**10**^−^** ^5^ M) was recorded in the range of 290–350 nm. The emission spectrum of histone (3.0**×**10**^−^** ^5^ M) was also recorded in the range of 290–350 nm. The overlap of the UV absorption spectrum of the EPI with the fluorescence emission spectrum of the histone core was used to determine the energy transfer as per the Forster’s theory [14].The efficiency of energy transfer, *E* is related to *R*, the distance between the donor and the acceptor by:
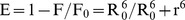
(7)where *F* and *F*_0_ are the fluorescence intensities of the protein in the presence and absence of the drug, *r* the distance between acceptor and donor and *R*_0_ is the critical distance when the transfer efficiency is 50%:
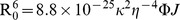
(8)
*k^2^* is the spatial orientation factor of the dipole, η the refractive index of the medium, Φ the fluorescence quantum yield of the donor and *J* is the overlap integral of the fluorescence emission spectrum of the donor and the absorption spectrum of the acceptor. *J* is approximated by the given equation:
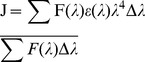
(9)where F(λ) is the fluorescence intensity of the fluorescent donor of wavelength, λ and ε(λ) is the molar absorption coefficient of the acceptor at wavelength, λ.

### Cell Culture and Drug Treatment

The experiments were carried out with the haploid yeast *Saccharomyces cerevisiae* strain from clontech laboratories (AH109) mating type *a* and Human embryonic kidney (HEK 293) were obtained from ATCC (CRL-1573). Yeast cells were grown YPD broth at 303 *K*. Cultures from exponential phase were diluted to a density of 10^7^ cells/ml and were incubated further with EPI concentrations for eight hours. HEK 293 cell (5 ×10^6^) at 70% confluency grown in DMEM medium were treated with different drug concentrations and used as required.

### Nuclear Extract Preparation

Nuclear extracts were prepared as described by Ponticelli *et al*. [41]. Briefly, yeast cells harvested and resuspended in YPD+1 M sorbitol, and digested with 150 mg of Zymolase 100T (Miles Laboratories, Inc.) at 303 *K* and the spheroplasts were recovered by centrifugation at 3,000**×**g for 5 min. The spheroplasts were lysed in 600 ml of buffer A (18% [wt/vol] Ficoll 400, 10 mM Tris hydrochloride [pH 7.5], 20 mM KCl, 5 mM MgCl_2_, 3 mM DTT, 1 mM EDTA, 0.5 mM spermidine, 0.15 mM spermine) containing lX protease inhibitors with a homogenizer. Cell debris and unlysed spheroplasts were removed by four sequential centrifugations. Nuclei were harvested by centrifugation at 25,000**×**g for 30 min and suspended in 45 ml of buffer B (0.1 M Tris acetate [pH 7.9], 50 mM potassium acetate, 10 mM MgSO4, 20% glycerol, 3 mM DTT, 2 mM EDTA, l × protease inhibitors). Aliquots were then incubated at 277*K* for 30 min, centrifuged at 21,000**×** *g*, and supernatant containing nuclear proteins was stored at −343*K*. Protein was estimated using Bradford method [42].

### Immunobloting

Equal amounts of proteins were loaded on 15% SDS-PAGE. Proteins were transferred to nitrocellulose membranes. Membranes were blocked with 5% milk in TBS-Tween 20 solution for 1 h at RT, followed by overnight incubation in primary antibody (anti-Acetyl histone K9/K14) at 277 *K* and subsequently incubated with secondary antibody conjugated with horseradish peroxidase (Sigma-Aldrich) for 1 hr at room temperature. Detection was done by ECL-Western Lightning Chemiluminescence reagent (Amersham Pharmacia). For re-probing membranes were stripped in 10% SDS buffer and probed for histone H3 to normalize for protein loading in each lane.

### Fourier Transform Infrared Spectroscopy (FTIR)

Infrared spectra of DNA solutions were recorded on a Nicolet Magna 750FT-IR spectrophotometer (DTGS detector, Ni-chrome source and KBr beam splitter) with resolution of 4 cm^−1^ and 60 scans. Spectra processing procedures included the spectra of sample solution and buffer collected under the same conditions followed by substraction of buffer or buffer+drug spectra from the sample spectra to obtain the FT-IR spectra of DNA.

### Electrophoretic Mobility Shift Assay (EMSA)

The electrophoretic mobility shift assay was performed by using the double-stranded synthetic oligonucleotides with the octamer consensus motif *ATGCAAAT*. The synthetic oligonucleotides were end-labelled using [γ^32^P] dATP and T4 polynucleotide kinase (MBI Fermentas). EPI-DNA reactions were set up with 0.1–6.0 μΜ of EPI and 0.25 ng ^32^P labelled oligonucleotides in a buffer containing 20 mM HEPES, 5 mM dithiothreitol, 7.5 mM MgCl_2_ and 5% glycerol. Reactions were incubated for up to 9 hrs. Nuclear extracts from HEK293 cells were then added to the reactions along with the nonspecific competitor poly (dI-dC): poly(dI-dC) [43] and the binding reaction was further incubated for 20 min at RT. Samples were electrophoresed at constant voltage (200 V) under low ionic strength conditions (0.25 M Tris/acetate/EDTA buffer) on 6% polyacrylamide gels. Gels were dried and subjected to phosphoimager for EPI-DNA detection.

### Yeast Two Hybrid Assay

The Matchmaker GAL-4-based yeast two-hybrid system (Clontech) was used. The yeast two-hybrid study was performed as described previously [44]. Briefly, AD-SNF4 (pSE1111) and DBD-SNF1 (pSE1112) hybrid vectors contained the SNF4 and SNF1 genes cloned in frame and downstream of the AD- and DBD- regions. Gene products of SNF4 and SNF1 have been shown to interact with each other using the yeast-two hybrid system. The constructs pSE1111 and pSE1112 were sequentially or cotransformed into the *S. cerevisiae* Y190 host strain. Transformed colonies grown in logarithmic phase were incubated with different concentration of EPI in the broth or the agar plate as required. Cells were assayed for β-galactosidase activity by both filter lift and liquid assays as described previously [45].

### Confocal Laser Scanning Microscopy (CLSM)

Confocal microscopy images were acquired to asses the effect of EPI on normal cell growth. A Leica TCS SP2 confocal system attached to a research Leica DM RXA2 microscope (Heidelberg, Germany) fitted with a water immersion dipping objective lens (60X) and a Kr-Ar Laser was utilized. The specimens were stained for 1 h with PI (0.2 mg/ml) in a buffer containing 0.1% sodium citrate, 0.1 mg/ml RNase and 0.3% Brij-58. The excitation wavelength was 594 nm. A scan speed of 400 lines s^−1^ was used to ensure minimum dislocation due to the movement of the cells. An HCX PL APO CS 63.0 × 1.32 Oil UV objective was used with an additional zoom of 4 x, resulting in a 512**×**512 image with a pixel size of 0.12 µm. The Kr-Ar lasers were used at 30% and 45% power, respectively, to minimize photobleaching. The images of control and test samples were averaged and compared.

### Membrane Integrity Assay

A spectrofluorimeter (Shimadzu, Japan), equipped with a 490-nm excitation filter was scanned 520–605 nm wavelength. The degree of membrane damage of EPI treated samples was assessed by PI binding to their cellular DNA in terms of fluorescence intensity units. Isopropanol (control) treated cells gave the highest fluorescence intensity reading; thus, fluorescence of all treatments was reported as a relative fluorescence of this control.

### Flow Cytometry

Samples (yeast cells) were stained by incubating with 20 mM PI for 15 min at 298 *K*. Flow cytometric analyses were done with a Coulter Epics Elite flow cytometer (Beckman Coulter France S.A., Paris, France). Typically, signals from 10,000 cells were acquired and analyzed for each sample using the Window Multiple Document Interface for Flow Cytometry (Win MDI) 2.8 software. The flow cytometry results presented in this study are representative of three independent experiments.

### Statistical Analysis

All readings were repeated at least three times and each experiment was performed at least in duplicate. The data were expressed as means ± SD (standard deviation), wherever required. Statistical analysis was performed by using Student’s *t* test. The criterion for statistical significance was *p*<0.05. The data obtained were analyzed statistically using SPSSv.10 software (SPSS Inc.).

## Supporting Information

Figure S1 Representative absorption spectra of DNA and EPI drug interaction. The curve a depit the native DNA and curve b-d, depits DNA-drug complex.
(TIF)

Figure S2 EMSA gel shift assay. The gel represents the probing of the presence of OCT1 protein in nuclear extract by supershift band. Lane 1: nucleat extract+probe+drug; lane 2: probe+drug and lane 3: nuclear extract+oligo probe+drug+anti-Oct1.
(TIF)

Figure S3 Effect of EPI treatment on cancerous cell line (SKM-1). Cells were treated with different concentration the drug and an untreated contol for 8 hrs and nuclear extract were prepared to probed the acetylation levels of histone H3 (Lysine 9/K9 and Lysine 14/K14). Total H3 protein was used as input control.
(TIF)

Figure S4 Molecular modeling of histone H3 binding with EPI, DOX and DNR. DNA is represented as orange color stick model whereas EPI, DOX and DNR are represented as red, yellow and blue color, respectively. Histone H3 shown in surface filled model cyan.
(TIF)

Figure S5 Immunoblot analysis of acetylation levels for histone H3 lysines K14, K18 and K23 on treatment with EPI for 8 hrs.Results show no effect of EPI on the acetylation level of the tested lysines.
(TIF)
