# Supplementary figures and images for: Inhibition of N-Terminal Lysines Acetylation and Transcription Factor Assembly by Epirubicin Induced Deranged Cell Homeostasis
Source: PLoS One. 2012 Dec 14;7(12):e51850. doi: 10.1371/journal.pone.0051850 (PMC3522591; doi:10.1371/journal.pone.0051850)

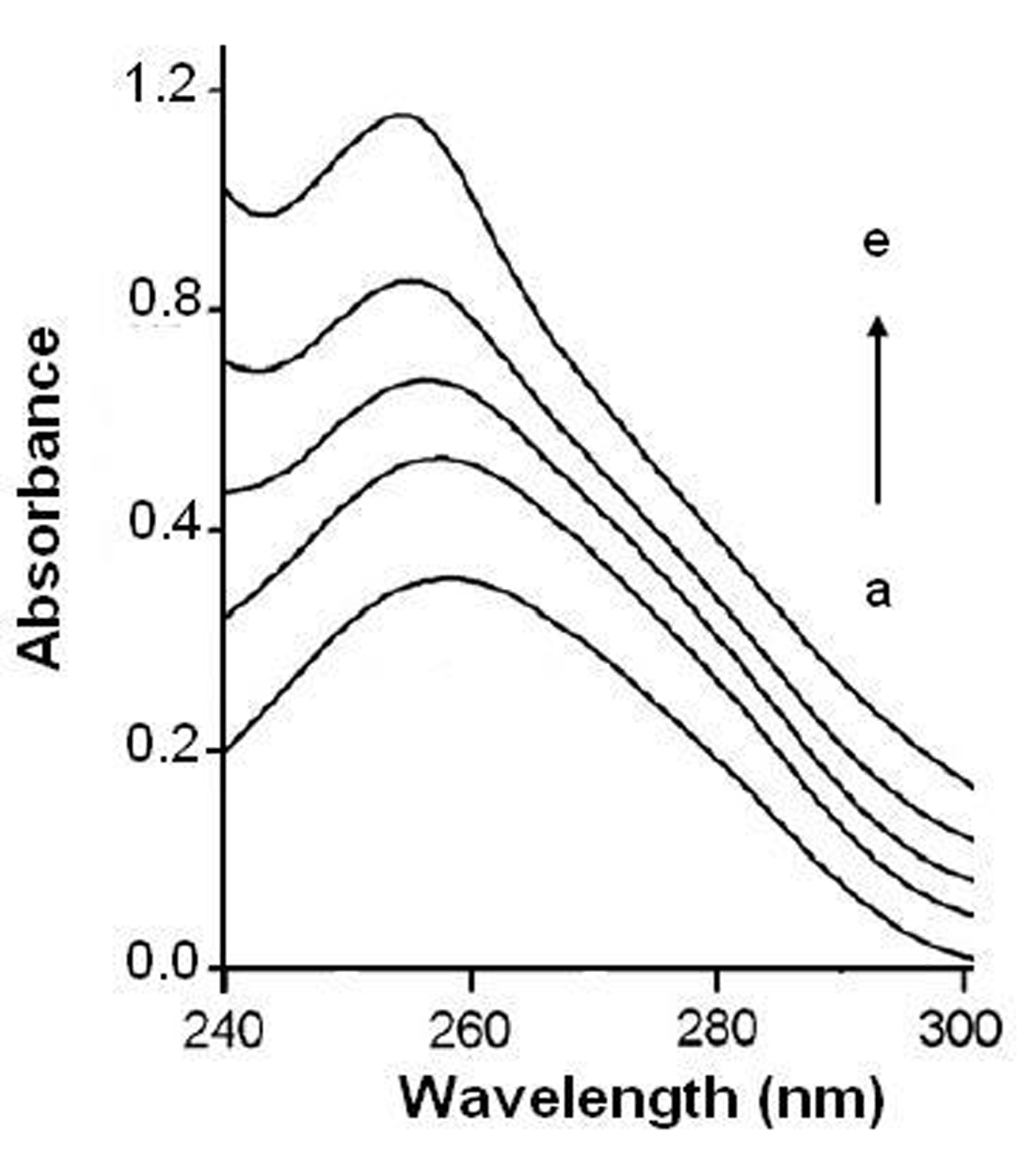

Supplement: Figure S1 — Representative absorption spectra of DNA and EPI drug interaction. The curve a depit the native DNA and curve b-d, depits DNA-drug complex. (TIF) [file pone.0051850.s001.tif]

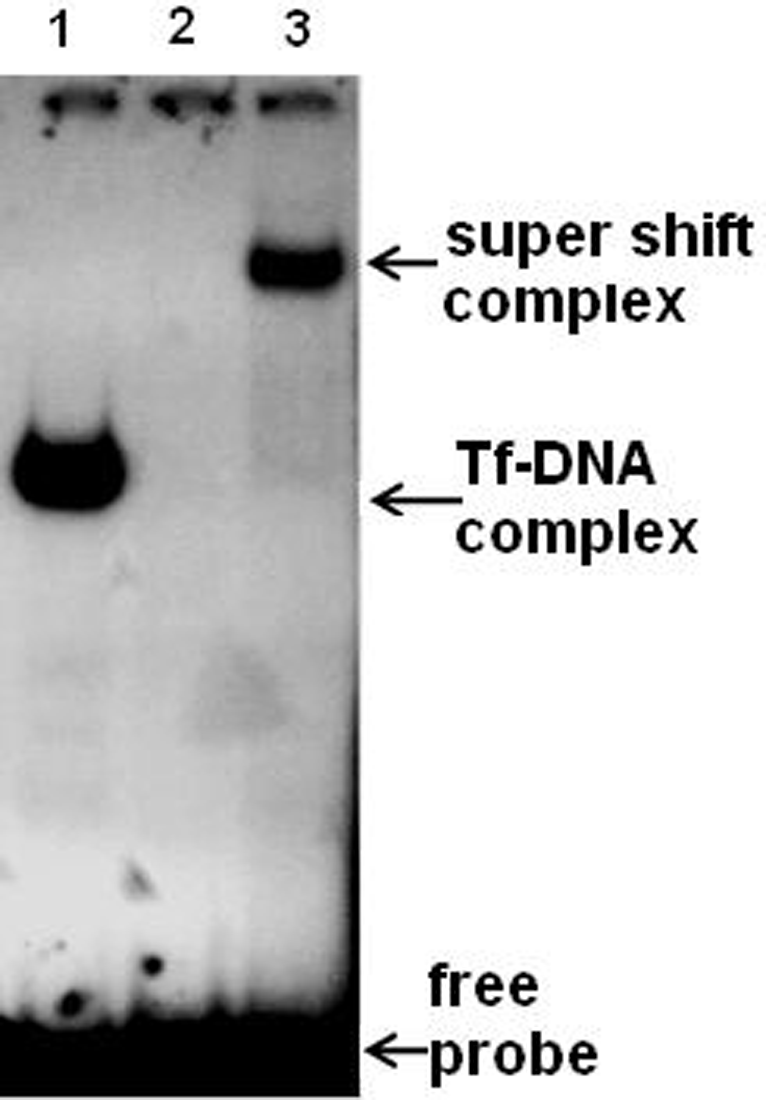

Supplement: Figure S2 — EMSA gel shift assay. The gel represents the probing of the presence of OCT1 protein in nuclear extract by supershift band. Lane 1: nucleat extract+probe+drug; lane 2: probe+drug and lane 3: nuclear extract+oligo probe+drug+anti-Oct1. (TIF) [file pone.0051850.s002.tif]

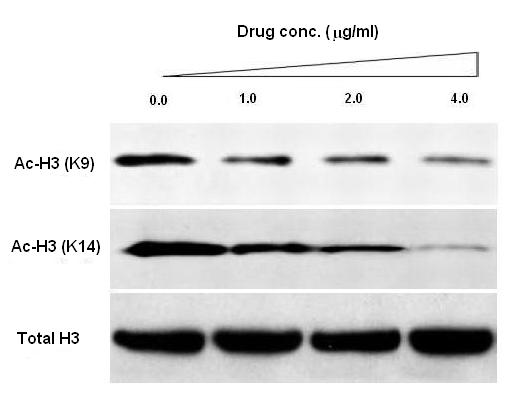

Supplement: Figure S3 — Effect of EPI treatment on cancerous cell line (SKM-1). Cells were treated with different concentration the drug and an untreated contol for 8 hrs and nuclear extract were prepared to probed the acetylation levels of histone H3 (Lysine 9/K9 and Lysine 14/K14). Total H3 protein was used as input control. (TIF) [file pone.0051850.s003.tif]

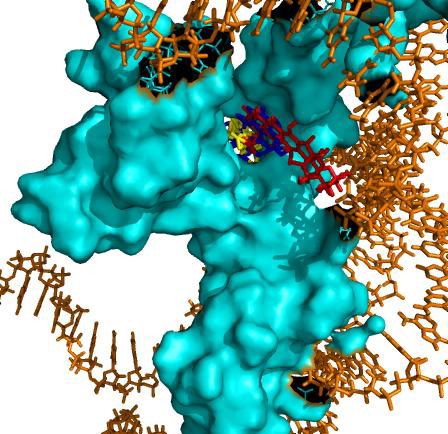

Supplement: Figure S4 — Molecular modeling of histone H3 binding with EPI, DOX and DNR. DNA is represented as orange color stick model whereas EPI, DOX and DNR are represented as red, yellow and blue color, respectively. Histone H3 shown in surface filled model cyan. (TIF) [file pone.0051850.s004.tif]

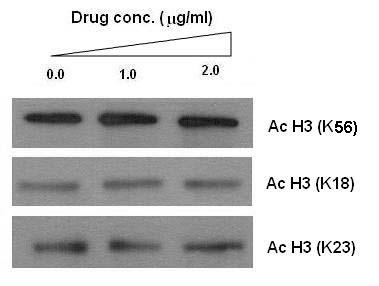

Supplement: Figure S5 — Immunoblot analysis of acetylation levels for histone H3 lysines K14, K18 and K23 on treatment with EPI for 8 hrs.Results show no effect of EPI on the acetylation level of the tested lysines. (TIF) [file pone.0051850.s005.tif]
